# Characterizing the Invasive Tumor Front of Aggressive Uterine Adenocarcinoma and Leiomyosarcoma

**DOI:** 10.3389/fcell.2021.670185

**Published:** 2021-06-03

**Authors:** Sabina Sanegre, Núria Eritja, Carlos de Andrea, Juan Diaz-Martin, Ángel Diaz-Lagares, María Amalia Jácome, Carmen Salguero-Aranda, David García Ros, Ben Davidson, Rafel Lopez, Ignacio Melero, Samuel Navarro, Santiago Ramon y Cajal, Enrique de Alava, Xavier Matias-Guiu, Rosa Noguera

**Affiliations:** ^1^Cancer CIBER (CIBERONC), Madrid, Spain; ^2^Department of Pathology, School of Medical, University of Valencia-INCLIVA, Valencia, Spain; ^3^Institut de Recerca Biomèdica de LLeida (IRBLLEIDA), Institut d’Investigació Biomèdica de Bellvitge (IDIBELL), Department of Pathology, Hospital U Arnau de Vilanova and Hospital U de Bellvitge, University of Lleida - University of Barcelona, Barcelona, Spain; ^4^Clínica Universidad de Navarra, University of Navarra, Pamplona, Spain; ^5^Institute of Biomedicine of Sevilla, Virgen del Rocio University Hospital/CSIC/University of Sevilla/CIBERONC, Seville, Spain; ^6^Cancer Epigenomics, Translational Medical Oncology Group (Oncomet), Health Research Institute of Santiago (IDIS), University Clinical Hospital of Santiago (CHUS/SERGAS), Santiago de Compostela, Spain; ^7^Department of Mathematics, MODES Group, CITIC, Faculty of Science, Universidade da Coruña, A Coruña, Spain; ^8^Institute of Clinical Medicine, Faculty of Medicine, University of Oslo, Oslo, Norway; ^9^Department of Pathology, Norwegian Radium Hospital, Oslo University Hospital, Oslo, Norway; ^10^Translational Medical Oncology Group (Oncomet), Health Research Institute of Santiago (IDIS), University Clinical Hospital of Santiago (CHUS/SERGAS), Santiago de Compostela, Spain; ^11^Roche-Chus Joint Unit, Translational Medical Oncology Group (Oncomet), Health Research Institute of Santiago (IDIS), Santiago de Compostela, Spain; ^12^Department of Pathology, Vall d’Hebron University Hospital, Autonomous University of Barcelona, Barcelona, Spain

**Keywords:** tumor-host interface, tumor microenvironment, extracellular matrix, reticular fibers, immune cells, gene expression, epigenetic profiles

## Abstract

The invasive tumor front (the tumor–host interface) is vitally important in malignant cell progression and metastasis. Tumor cell interactions with resident and infiltrating host cells and with the surrounding extracellular matrix and secreted factors ultimately determine the fate of the tumor. Herein we focus on the invasive tumor front, making an in-depth characterization of reticular fiber scaffolding, infiltrating immune cells, gene expression, and epigenetic profiles of classified aggressive primary uterine adenocarcinomas (24 patients) and leiomyosarcomas (11 patients). Sections of formalin-fixed samples before and after microdissection were scanned and studied. Reticular fiber architecture and immune cell infiltration were analyzed by automatized algorithms in colocalized regions of interest. Despite morphometric resemblance between reticular fibers and high presence of macrophages, we found some variance in other immune cell populations and distinctive gene expression and cell adhesion-related methylation signatures. Although no evident overall differences in immune response were detected at the gene expression and methylation level, impaired antimicrobial humoral response might be involved in uterine leiomyosarcoma spread. Similarities found at the invasive tumor front of uterine adenocarcinomas and leiomyosarcomas could facilitate the use of common biomarkers and therapies. Furthermore, molecular and architectural characterization of the invasive front of uterine malignancies may provide additional prognostic information beyond established prognostic factors.

## Introduction

Uterine endometrial adenocarcinoma (uADC) is the fourth most common cancer among women in the Western world, with an estimated incidence of 10–20 per 100,000 women. Although prognosis is favorable for patients identified with low-grade tumors and early-stage disease, outcomes for patients with high-grade and metastatic/recurrent tumors remain poor (Ferlay et al., 2010) and represent a therapeutic challenge. The World Health Organization (WHO) tumor classification distinguishes several histopathological types of uADC, particularly based on microscopic appearance (Cree et al., 2020): (1) endometrioid carcinoma, low grade (grades I–II) or high grade (grade III), (2) serous carcinoma, (3) clear cell carcinoma, (4) mixed carcinoma, (5) undifferentiated carcinoma, (6) carcinosarcoma, (7) neuroendocrine carcinomas, and (8) other unusual types. These types have different histological and molecular features, precursor lesions, and natural history (Matias-guiu et al., 2001; Yeramian et al., 2013). They are also stratified by Tumor Cancer Genome Atlas (TCGA)-based molecular classification into four risk groups combining gene encoding DNA polymerase ε (POLE) mutational analysis with IHC analysis of p53 and mismatch repair (MMR) proteins (PMS-2 and MSH-6) (Getz et al., 2013). This provides additional prognostic information to complement the microscopic features.

Uterine leiomyosarcoma (uLMS) accounts for 1% of all uterine malignancies, with an annual incidence rate of 0.4–0.9 per 100,000 women. Like other forms of sarcoma, uLMS spreads to other parts of the body via the bloodstream rather than the lymphatic system. Although rare, this cancer can be extremely aggressive and is known to be generally unresponsive to radiation or chemotherapy. Patient survival is highly dependent on speed of diagnosis and treatment, falling to 14% at 5 years post-diagnosis for metastatic uLMS. From a molecular viewpoint, uLMS contains complex karyotypes, with numerous chromosomal aberrations and frequent deletions affecting chromosomal arms 2p, 2q, 10q, and 13q, as well as amplifications on 1p, 5q, and 8q, and precise alterations in TP53, RB1, PTEN, MED12, YWHAE, and VIPR2 (Cuppens et al., 2018).

There is growing interest in understanding the molecular features involved in myometrial invasion, a highly valuable parameter due to its direct association with poor prognosis and limited therapeutic response in uADC and uLMS. From a histopathological perspective, a variety of invasion patterns frequently coexist in different areas of the same tumor (Euscher et al., 2013). Moreover, several tumor types show specific invasion patterns not seen in other types of cancer. These differences can be explained by the fact that cancer cell invasion is currently viewed as an adaptive and heterogeneous process (Friedl and Alexander, 2011) involving key processes such as cytoskeleton dynamics, cell adhesion plasticity, and mechanotransduction of external stimuli.

Type-specific new stroma generated at the site of active tumor invasion, the invasive tumor front (ITF), is crucial in tumor growth and invasion processes (Provenzano et al., 2006; Giatromanolaki et al., 2007). In fact, high proliferation rates and cell cycle derailment have been shown at the ITF in uADC (Horrée et al., 2007). Structures surrounding tumors such as mature collagen and smooth or striated muscle can act as a barrier to tumor invasion, as shown by collagen organization at the ITF of oral squamous cell carcinomas (Devendra et al., 2018). However, tumor cells can disrupt the continuity of such structures by remodeling the immediate stroma of the tumor to carve out paths for invasion. Indeed, invasive tumor growth in pancreatic ductal adenocarcinoma is mediated via matrix remodeling metalloproteinases (Croft et al., 2004; Rath et al., 2017). Changes in the fibrillar pattern of adjacent stroma of tumor tissues have been also observed in skin tumor (Stenbäck et al., 1999), oral squamous cell carcinoma, and lymph nodes with metastasis, among others (Yinti et al., 2015; Kardam et al., 2016). In fact, the tumor-derived extracellular matrix (ECM) is biochemically distinct in its composition and is stiffer than normal ECM. This new associated ECM compartment, rich in cross-linked collagen III (reticular fibers), has been proposed as a prospective marker of early stromal invasion in incipient tumors such as breast cancer (Sivridis et al., 2004; Acerbi et al., 2015). In addition, not only the ECM collagen composition but also the orientation of the fibers have been proposed as a prognostic signature for survival in breast cancer (Conklin et al., 2011; Xi et al., 2021).

The complex interaction between heterogenic immune cell subpopulations and tumor cells may drive tumor progression, metastasis, and resistance to therapy (Galli et al., 2020). The intensifying development of immunotherapeutic strategies calls for a better understanding of tumor-immune subpopulation interactions and spatial distribution at the ITF (Blomberg et al., 2018; König et al., 2019). In this context, the use of a multiplex immunolabeling panel is essential as it enables different cell subpopulations to be identified in one tissue section (Gorris et al., 2018). Multiplexed analysis can also simultaneously measure the expression of distinct markers in a single cell as well as spatial associations between immune cell subpopulations. We applied this technology to evaluate the complex immune environment of the ITF of uADC and uLMS. A previously developed and validated multiplex immunolabeling panel was used to simultaneously assess the phagocytic cell marker CD68 of macrophages, CD3 + and CD8 + T cells, and CD20 + B lymphocytes in a single FFPE tissue (Abengozar-Muela et al., 2020; Salas-Benito et al., 2021).

Changes in activated-leukocyte cell adhesion molecule (ALCAM) expression at the endometrial tumoral cell surface (Devis et al., 2018) and increased expression of cytoplasmic Cyclin D1 (Fusté et al., 2016) have been reported to allow dissemination and invasion of endometrial neoplastic cells. Epigenetic mechanisms play an important role in regulating gene expression during many biological processes (Sharma et al., 2009). DNA methylation is the most widely studied epigenetic modification, produced by adding a methyl group (CH3) to the 5’ carbon of cytosines in cytosine–phosphate–guanine (CpG) dinucleotides to generate 5-methylcytosine (5mC) (Bao-Caamano et al., 2020). Deregulation of this epigenetic mechanism has major implications for cancer development and progression (Diaz-Lagares et al., 2016). In this context, recent genome-wide analyses have revealed striking alterations in the methylation profile of uterine uADC and uLMS (Zhang et al., 2014; Kommoss et al., 2020; Vargas et al., 2021).

Cancers show a clear coevolution between tumor cells and the tumor microenvironment. Specific differences in the tumor microenvironment at different locations may play a role in tumor growth, metastatic progression, and therapy responses (Oliver et al., 2018; Zhang and Yu, 2019). In fact, tumor invasion is a dynamic process facilitated by bidirectional interactions between tumor cells and the microenvironment, being particularly intense at the ITF. Unlike tumor cells, tumor microenvironment elements are genetically stable and thus represent an attractive therapeutic target. Uterine uLMS and uADC are different tumor types occurring in the same organ, both of which infiltrate the myometrium during local progression. The main objective of the present study is to analyze several aspects of microenvironment response at the ITF in these two tumor types, looking for similarities and differences that could provide insight into potential new common therapeutic approaches, an aim that to our best knowledge has not previously been addressed.

## Materials and Methods

### Patients, Sample Description, and Case Selection

A search for uADC and uLMS cases was conducted in the study institutions. Selection criteria included available pathology reports, representative sections of the ITF, histologically proven distant metastasis, and acceptable pre-analytical conditions. Cases were reviewed by a panel of expert gynecological pathologists from the institutions involved. In total, 24 uADC and 11 uLMS fulfilled all criteria and were included in the study.

The study used formalin-fixed and paraffin-embedded (FFPE) tissue samples from 24 uADC and 11 uLMS obtained from five Spanish hospitals (Hospital Clínico de Valencia; Hospital Virgen del Rocio, Seville; Hospital Universitari Vall d’Hebron and Hospital Universitari de Bellvitge, Barcelona; Hospital Universitari Arnau de Vilanova, Lleida) and the Norwegian Radium Hospital, Oslo University Hospital, Oslo, Norway. Tumors were classified following the most recent WHO criteria and were surgically staged and graded according to the FIGO (International Federation of Gynecology and Obstetrics) staging and grading systems. The study was approved by the local research ethics committee, and specific informed consent was obtained.

Whole slide FFPE tissue sections of 5 μm of selected uADC and uLMS were stained with H&E (hematoxylin and eosin) and examined by the centralized expert group of pathologists to select the representative areas to include in the study. The interface between tumor tissue and adjacent myometrium was microdissected under the microscope. Microscopic images were obtained using a digital slide scanner [Pannoramic 250 FLASH II 2.0 (3D Histec)].

All uADC were of endometrioid type and were classified according to the Cancer Genome Atlas (TCGA) surrogate (0% POLE mutated, 58.5% non-specific molecular profile, 29% mismatch repair-deficient tumors, and 12.5% p53 abnormal). All uLMS were conventional-type high-grade tumors.

### Selection of Regions of Interest

Sections of FFPE tissue samples were scanned before and after microdissection. Whole sections including the interface between tumor tissue and adjacent myometrium areas (the ITF) were used for morphometric analysis. ITF microdissected tissue was employed for transcriptomic and epigenomic studies (Supplementary Figure 1A). The amount of ITF microdissected tissue varied from case to case, but the median width was 5 mm (±1.66 mm) and median length 15 mm (±4.91 mm). The amount of tumor vs. myometrium is shown in Supplementary Table 1.

Serial uADC and uLMS whole slides were used for histomorphometric analysis of reticular fibers and multiplex immunofluorescence-based immune profiling. ITF regions were identified in stained tissue (Gomori and multiplex immunofluorescence) by extrapolation of previous H&E-selected regions. A region of interest (ROI) of 5 × 4 mm for each sample was identified within the ITF-stained area. These 5 × 4-mm regions were used to correlate the results between genomic, epigenetic, and morphometric studies (Supplementary Figure 1A). To achieve in-depth characterization of the reticular fibers and immune infiltrate of the ITF, we further broke down the 5 × 4-mm ROIs into 1-mm^2^ ROIs representing the following categories: (a) tumor (uADC or uLMS with absence of myometrium), (b) myometrium (excluding infiltrated myometrium from the analysis), and (c) balanced representation of the invasion front (containing 50% tumor and 50% myometrium). Supplementary Figure 1 shows a schematic representation of the procedure. Cases and ROIs where the algorithm failed because of poor or excessive staining were excluded from the analysis, as were samples with unsatisfactory segmentation. The number of 5 × 4-mm and 1-mm^2^ ROIs of each case in Gomori and multiplex immunofluorescence stained tissue are shown in Supplementary Table 1.

### Histomorphometric Analysis of Reticular Fibers

The architecture of uADC and uLMS reticular fibers stained using Gomori’s method was uncovered. All samples were digitized with the whole-slide scanner Ventana iScan HT (Roche) at 20 × with a resolution of 0.46 μm/pixel. We used the open-source digital pathology software QuPath for sample visualization and identification of ROIs (Bankhead et al., 2017). 5 × 4-mm and 1-mm^2^ ROIs were exported to ImageJ (Schneider et al., 2012), and these were saved as TIFF for image analysis.

In this study, we employed an advanced morphometric methodology based on a probabilistic method for the automatic segmentation of reticular fibers. We used Gomoripath, a morphometric tool for easy segmentation of reticular fibers developed by researchers from Incliva Biomedical Research Institute/University of Valencia, University of Castilla La Mancha, and the Andalucía Public Health System (Espinosa-Aranda et al., 2016). Briefly, the algorithm is based on nominal logistic regression applied to the optical density of the histopathological images. The optical density is calculated iteratively, allowing reticular fibers to be enhanced. Subsequently, a logit model is used to calculate the probability of each pixel belonging to the structure of reticular fibers depending on their topology, thus creating a probability map for the entire image. The ROC (Receiver Operating Characteristic) curve generated by the model finally obtained an AUC (area under the ROC curve) of 0.9563 in reticular fiber detection.

**FIGURE 1:**
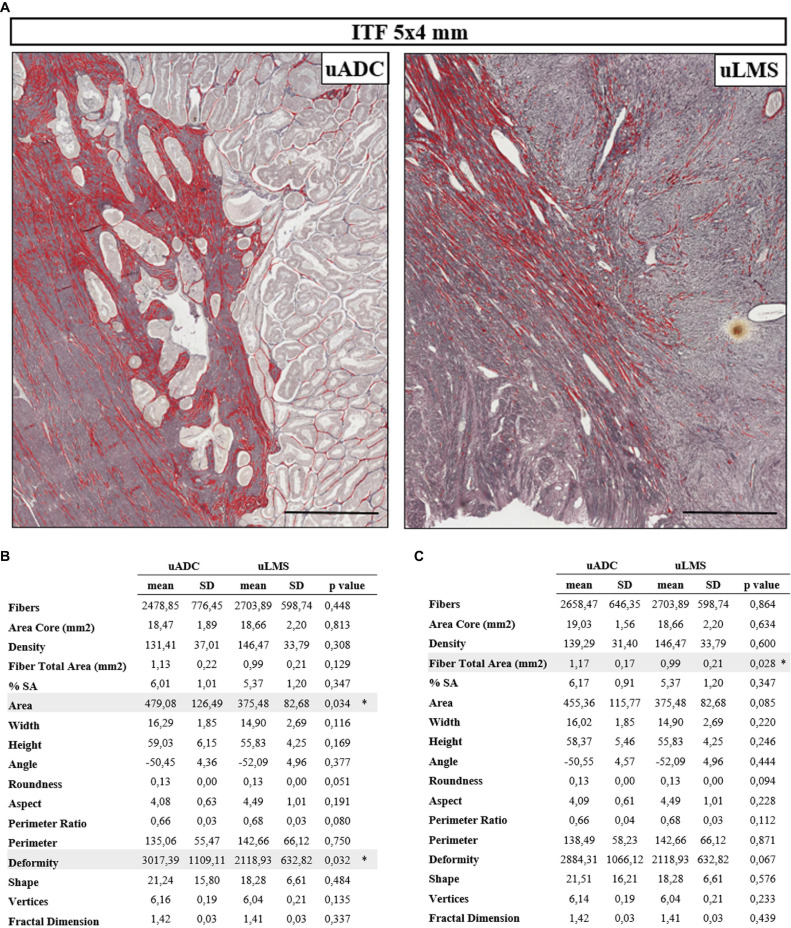
Histomorphometric features of reticular fibers in uterine adenocarcinomas (uADC) and leiomyosarcomas (uLMS) in the 5 × 4-mm region of interest (ROI) are very similar at the invasive tumor front (ITF). **(A)** Representative image of fiber segmentation in 5 × 4-mm ITF ROIs in uADC and uLMS. Reticular fibers are highlighted in red. Scale bar represents 1 mm. **(B)** Comparison of morphometric parameters obtained after reticular fiber segmentation between uADC and uLMS 5 × 4-mm ITF ROIs. Fisher–Snedecor test and Student *T*-test were applied for statistical comparison. Mean, standard deviation (SD), *p*-Values, and significance (^∗^ < 0.05) are shown. Total fibers, area of the core, and sum of the total area of the fibers were measured to calculate the density (number of fibers/mm^2^) and percentage of the stained area (% SA). Area in μm^2^. Length and width in μm. **(C)** The same comparison as in B with more restrictive criteria for case selection.

**FIGURE 2:**
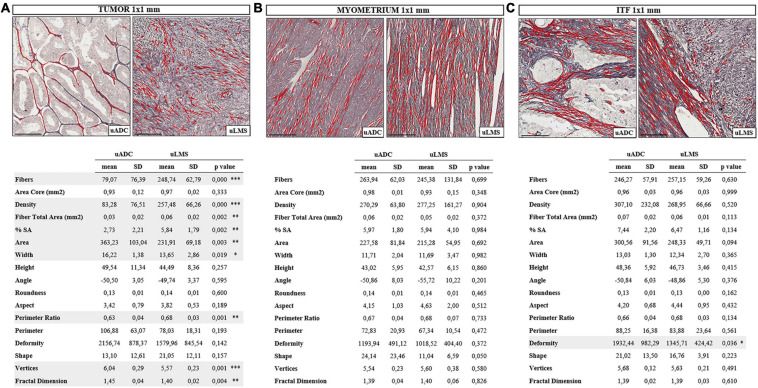
In-depth invasive tumor front (ITF) histomorphometric characterization of reticular fibers in uterine adenocarcinomas (uADC) and leiomyosarcomas (uLMS) reveals a high degree of architectural similarity. Representative image of fiber segmentation in 1 × 1-mm tumor ROIs in uADC and uLMS. Comparison of morphometric parameters obtained after reticular fiber segmentation between uADC and uLMS in panels **(A)** Intratumor 1 × 1-mm ROIs. **(B)** 1 × 1-mm myometrium ROIs, and **(C)** 1 × 1-mm invasive tumor front (ITF) ROIs in uADC and uLMS. Reticular fibers are highlighted in red. Fisher–Snedecor test and Student *T*-test were applied for statistical comparison. Mean, standard deviation (SD), *p*-Values, and significance (^∗^ < 0.05, ^∗∗^ < 0.01, ^∗∗∗^ < 0.001) are shown. Total fibers, area of the core, and sum of the total area of fibers were measured to calculate the density (number of fibers/mm^2^) and percentage of stained area (% SA). Area in μm^2^. Length and width in μm. Scale bars represent 250 μm.

**FIGURE 3:**
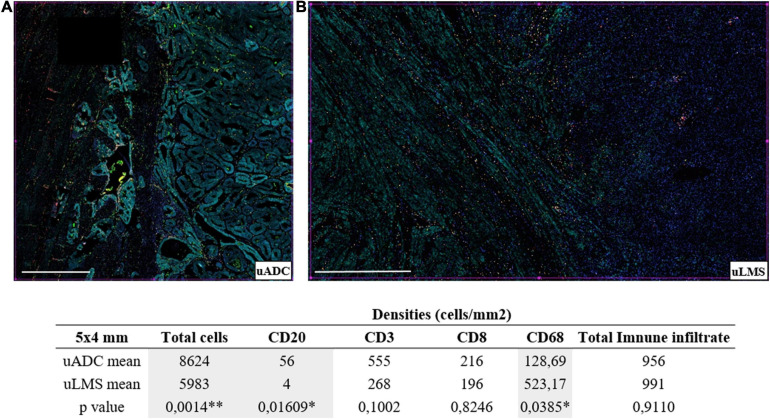
Distinct immune infiltrate distribution at the invasive tumor front (ITF) of uterine adenocarcinomas (uADC) and leiomyosarcomas (uLMS). **(A)** Representative multiplex immunofluorescence images of approximately 5 × 4-mm regions of interest (ROIs) of an adenocarcinoma (uADC) (4 × 4) and a leiomyosarcoma (uLMS) (5 × 3 mm). Color code as follows: DAPI (blue), CD20 (yellow), CD68 (green), CD8 (red), CD3 (orange), cytokeratin (cyan). **(B)** The mean of the densities (number of cells/mm^2^) for each cell marker is shown for uADC and uLMS as well as the mean for the total cells and the total immune infiltrate. Fisher–Snedecor test and Student *T*-test were applied for statistical comparison. *P*-values and significance (^∗^ < 0.05, ^∗∗^ < 0.01) for statistical comparison between the means of uADC and uLMS are displayed. Scale bars represent 1 mm.

Fifteen morphometric parameters defining the histological organization of reticular fibers were calculated for each fiber detected and the mean for each sample calculated. Morphometric parameters were extracted to characterize the size and shape of the morphometric variables at the ITF. In addition, the algorithm measured the stained area of the tissue analyzed (excluding holes and damaged tissue), allowing us to determine the number of fibers per mm^2^ (density) and the percentage of fiber-stained area (%SA) (taking into account the sum of the areas of all fibers). Morphometric parameters defining the histological organization of the reticular fiber networks have been explained elsewhere (Tadeo et al., 2016). The mean of each parameter of similar uADC and uLMS ROIs was calculated for comparison (Figures 1–3).

### Multiplex Immunofluorescence Cell Phenotype

We next sought to investigate the uADC and uLMS myeloid and lymphocytic contexture in the FFPE tissue samples. A multiplex immunofluorescence panel was used to enable simultaneous examination of several cellular markers, including the phagocytic cell marker CD68 of macrophages, CD3 + and CD8 + T cells, and CD20 + B lymphocytes. Multiplex immunofluorescence development and validation workflow and protocols were implemented as previously described (Schalper et al., 2019; Abengozar-Muela et al., 2020; Salas-Benito et al., 2021). Briefly, 5-μm sections of FFPE tissue were deparaffinized and antigen retrieval was performed using DAKO PT-Link heat-induced antigen retrieval with low pH (pH 6) or high pH (pH 9) target retrieval solution (DAKO). Each tissue section was subjected to five successive rounds of antibody staining, each round consisting of protein blocking with antibody diluent/block (Akoya Biosciences ARD1001EA) and incubation with a primary antibody, Opal Polymer anti-mouse/rabbit HRP (Akoya Biosciences ARH1001EA), followed by tyramide signal amplification (TSA) with Opal fluorophores (Akoya Biosciences) diluted 1:100 in 1 × plus amplification diluent (Akoya Biosciences FP1498). The myeloid and lymphoid cell panel included CD68 (Mouse monoclonal, clone PG-M1, ready-to-use, Agilent IR613), CD3 (Rabbit polyclonal, IgG, ready-to-use, Agilent IR503), CD8 (Mouse monoclonal, clone C8/144B, ready-to-use, Agilent IR623), CD20 (Mouse monoclonal, IgG2α, clone L26, ready-to-use, Roche 760-2531), and cytokeratin (Mouse monoclonal, clone AE1/AE3, diluted 1:100, Agilent M3515). Finally, in the last round, nuclei were counterstained with spectral DAPI (Akoya Biosciences FP1490) and sections mounted with Faramount Aqueous Mounting Medium (Dako S3025).

**FIGURE 4:**
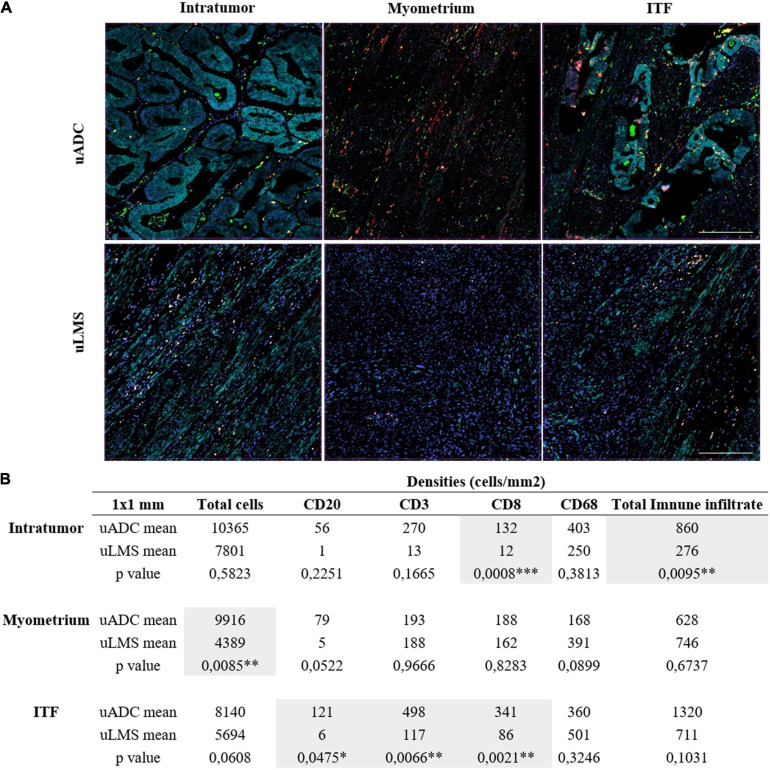
Immune infiltrate characterization in 1 × 1-mm regions of interest (ROIs) in uterine adenocarcinomas (uADC) and leiomyosarcomas (uLMS). **(A)** Representative multiplex images of 1 × 1-mm ROIs of intratumor, myometrium, and invasive tumor front (ITF) in uADC and uLMS. Color code as follows: DAPI (blue), CD20 (yellow), CD68 (green), CD8 (red), CD3 (orange), cytokeratin (cyan). **(B)** The mean of the densities (number of cells/mm^2^) for each cell marker in each region is shown for uADC and uLMS as well as the mean for the total cells and the total immune infiltrate. Fisher–Snedecor test and Student *T*-Test were applied for statistical comparison. *P*-values and significance (^∗^ < 0.05, ^∗∗^ < 0.01, ^∗∗∗^ < 0.001) for statistical comparison between the means of intratumor, myometrium, and ITF in uADC and uLMS are displayed. Scale bars represent 250 μm.

Each whole-tissue section was scanned on a Vectra-Polaris Automated Quantitative Pathology Imaging System (Akoya Biosciences). Tissue imaging and spectral unmixing were performed using InForm software (version 2.4.8, Akoya Biosciences), as previously described (Abengozar-Muela et al., 2020; Salas-Benito et al., 2021). Image analysis was performed on 5 × 4-mm and 1-mm^2^ ROIs using the open-source digital pathology software QuPath version 0.2.3, as previously described (Abengozar-Muela et al., 2020). In short, cell segmentation based on nuclear detection was performed on QuPath using the StarDist 2D algorithm, a method that localizes nuclei via star-convex polygons, incorporated into QuPath software by scripting. A random-tree algorithm classifier was trained separately for each cell marker by an experienced pathologist annotating the tumor regions. Interactive feedback on cell classification performance is provided during training in the form of mark-up image, significantly improving the accuracy of machine learning-based phenotyping (Bankhead et al., 2017; Abengozar-Muela et al., 2020). All phenotyping and subsequent quantifications were performed blinded to the sample identity. Cells close to the border of the images were removed to reduce the risk of artifacts. Based on the fluorescence panels, cells were further subclassified as CD68+, CD3+, CD8+, and CD20+. Cells negative for these markers were defined as other cell types. The mean of the frequency of each cell marker of similar uADC and uLMS ROIs was calculated for comparison (Figures 3, 4).

### Transcriptomic Profiling

Total RNA for gene expression assays was prepared from 5-μm FFPE tissue sections of microdissected ITF using the Agencourt FormaPure kit (A33341; Beckman Coulter, Indianapolis, IN, United States) and following the manufacturer’s instructions. The RNA concentration was determined with Qubit 4 Fluorometer and Qubit^®^ RNA HS Reagent (Thermo Fisher Scientific, Waltham, MA, United States). RNA samples passing the quality control evaluation (uADC *n* = 19, uLMS *n* = 11) were selected.

Transcriptomic profiling was performed with HTG EdgeSeq Precision Immuno-Oncology Panel, which interrogates 1,392 genes involved in tumor/immune interaction^1^. HTG EdgeSeq Chemistry was employed to synthetize the RNA-Seq library. Briefly, target capture was performed by hybridizing the mRNA with Nuclease Protection Probes (NPPs). The S1 nuclease was added to the mix, producing a stoichiometric amount of target mRNA/NPP duplexes. This reaction was blocked by enzyme heat denaturation of S1. The samples were randomized before inclusion in the HTG EdgeSeq system to reduce potential biases in the run. Each hybridized sample was used as template to set up PCR reactions with specially designed tags, sharing common sequences that are complementary to both 5′-end and 3′-sequences of the probes, and common adaptors required for cluster generation on an Illumina sequencing platform. In addition, each tag contains a unique barcode used for sample identification and multiplexing. After PCR amplification, a cleanup procedure was performed using Agencourt AMPure XP (Beckman Coulter). The library was quantified by quantitative PCR, using KAPA Library Quantification (Roche), according to the manufacturer’s instructions. All samples and controls were quantified in triplicate, and no template control was included in any run. Library denaturation was performed by first adding 2N NaOH, followed by addition of 2N HCl. The PhiX was spiked in at 5% (concentration of 12.5 pM). Normalized libraries were sequenced by NGS. Four demultiplexed FASTQ file per sample was retrieved from the sequencer for data processing. HTG EdgeSeq host software performed the alignment of the FASTQ files to the probe list, then results were parsed, and the output obtained as a read count matrix.

Raw count normalization and differential expression analysis were calculated using DESeq2 R package (1.30.0). Sample outliers were identified through variance stabilizing transformation. iDEP v0.92^2^ was used for pathway analysis of normalized expression values from RNA-Seq data (Ge et al., 2018).

### Genome-Wide DNA Methylation Analysis

Total genomic DNA from 10-μm FFPE tissue sections of microdissected ITF (uADC *n* = 24, uLMS *n* = 11) was isolated using the AllPrep DNA/RNA FFPE Kit (Qiagen) and following the manufacturer’s instructions. All DNA samples were quantified by the fluorometric method using the Qubit 1 × dsDNA HS (High-Sensitivity) Assay Kit (Thermo Fisher) and were also checked for suitability for FFPE restoration following the Infinium HD FFPE QC Assay (Illumina). DNA samples (100–250 ng) that passed this quality control evaluation (uADC *n* = 22, uLMS *n* = 9) were selected for bisulfite conversion using the EZ DNA Methylation kit (Zymo Research) and were moved on to the FFPE Restore protocol (Illumina). The restoring step was followed by Infinium HD FFPE methylation assay for hybridization with Infinium MethylationEPIC BeadChips, which cover over 850,000 CpG sites along the human genome (Moran et al., 2016). Whole-genome amplification and hybridization were performed on the BeadChips followed by single-base extension and analysis on a HiScan (Illumina) to assess the cytosine methylation states. Image intensities were extracted using GenomeStudio (V2011.1) Methylation module (1.9.0) software from Illumina. Data quality control was assessed with GenomeStudio and BeadArray Controls Reporter, based on the internal control probes present on the array. The methylation score of each CpG from samples that passed this quality control (uADC *n* = 21, uLMS *n* = 9) was represented as β-value and previously normalized for color bias adjustment, background level adjustment, and quantile normalization across arrays. Probes and sample filtering involved a two-step process for removing SNPs and unreliable β-values with a high detection *P*-value > 0.01. After this filtering step, the remaining CpGs were considered valid for the study. Non-parametric Wilcoxon tests were applied to determine differentially methylated CpGs (DMCpGs), which were considered significant with a false discovery rate (FDR) below 5%. All statistical analyses were performed in the R statistical environment (v.3.6.1). The enrichment analysis of biological pathways for the methylation profiles were evaluated by gene ontology (GO) using GENECODIS (Tabas-Madrid et al., 2012).

## Results

### Reticulin Fiber Scaffolding in uLMS and uADC Is Similar

Several parameters of reticular fibers were assessed in 83.3% of uADC and 81.8% of uLMS (20 of 24 uADC and 9 of 11 uLMS) (Supplementary Table 1). Interestingly, we found a high degree of similarity between the 5 × 4 mm ROIs of uADC and uLMS ITFs (Figures 1A,B); in fact, the only significant differences observed in the cases studied were in area size and deformity of the individual or meshwork fibers. However, we next performed a more restrictive analysis, excluding one case of each group based not only on algorithm quality control but on subjective assessment by two independent scientists (Figure 1C). In this case, we analyzed a more homogeneous sample group and observed no significant size or shape-related differences between the two tumor types. Large uADC ROIs with reticular fibers occupying a higher proportion of stained area (higher %SA) than uLMS were detected.

To test the robustness of the algorithm and assess the representativeness of the 1-mm^2^ ROIs, we compared four ROIs of 1 mm^2^ inside the 5 × 4 mm against four ROIs of 1 mm^2^ along the ITF for both uADC and uLMS. We found no significant differences between ROIs (data not shown) and therefore accepted the 1 × 1-mm areas as representative of the tumor, myometrium, and selected field of ITF. Only regions meeting the quality control parameters were included in the analysis. In total, we compared *n* = 21 vs. *n* = 34 tumor ROIs, *n* = 39 vs. *n* = 24 myometrium ROIs, and *n* = 79 vs. *n* = 35 selected ITF ROIs of uADC and uLMS, respectively (Supplementary Table 1). We employed the same morphometric feature extraction procedure as above, also performing the statistical Student test to compare the mean of each acquired parameter by patient sample. In the 1-mm^2^ ITF ROIs, the two tumor types presented a high number of significantly different parameters (7/15) (Figure 2A). uADC fibers appeared larger (area = 363.2 μm^2^ vs. 231.9 μm^2^) and thicker (width = 16.21 μm vs. 13.6 μm) than in uLMS. However, because uADC has significantly lower density of fibers per μm^2^ (83.2 vs. 257.48 fibers/μm^2^) when compared to uLMS, a smaller%SA of the tissue (2.73% vs. 5.84%) was shown. Furthermore, uADC reticular fibers appeared smoother than the wavy ones in uLMS, as indicated by the perimeter ratio (0.62 vs. 0.68). The higher values for vertices in uADC compared to uLMS (6.04 vs. 5.56) suggest that reticular fibers have greater branching in uADC. Finally, the fractal dimension revealed that reticular fibers are more haphazardly arranged in uADC than in uLMS (1.44 vs. 1.40). On the other hand, the myometrium of uADC and uLMS displayed no significant differences in any of the parameters studied (Figure 2B). Interestingly, only one shape parameter (deformity) was significantly different between uADC vs. uLMS (1932.4 vs. 1345.7) (Figure 2C). This observation is a more reliable indication than the 5 × 4-mm ROI results of a high degree of similarity between the reticular fiber scaffolding at the ITF of uADC and uLMS.

To further characterize uLMS and uADC, the tumor, myometrium, and selected ITF were compared against each other for each tumor type. In contrast to the great similarity between 1-mm^2^ ROIs of different tissue areas in uLMS, we found that uADC exhibited multiple differences when ITF was compared against the tumor or myometrium (Table 1). There was higher deposition of fibers, indicated by increased fiber density, in ITF compared to tumor (307.10 vs. 83.28), leading to an increased %SA (7.44 vs. 2.73). Reticular fibers at the uADC also appeared thinner at ITF than in tumor (width = 13.03 vs. 16.22), were linearized (aspect = 4.20 vs. 3.42), and presented less branching (vertices = 5.68 vs. 6.04). Finally, when comparing 1-mm^2^ ROIs of uADC ITF vs. myometrium, the fibers appeared bigger at the ITF (area = 300.56 vs. 270.29), were thicker (width = 13.03 vs. 11.71) and longer (height = 48.36 vs. 43.02), and therefore had a larger perimeter (88.25 vs. 72.83). In addition, the fibers at the uADC ITF appeared less linearized (aspect = 4.20 vs. 4.15) and branched (vertices = 5.68 vs. 5.54) (Table 1A). Interestingly, in uLMS the only significant difference when comparing both ITF and tumor vs. myometrium was fiber shape (shape ITF = 16.76 and tumor 21.05 vs. myometrium 11.04) (Table 1B).

**TABLE 1:** Heterogeneous architecture of reticular fibers between 1 × 1-mm regions of interest (ROIs) of tumor, myometrium, and invasive tumor front (ITF) within uterine adenocarcinomas (uADC).

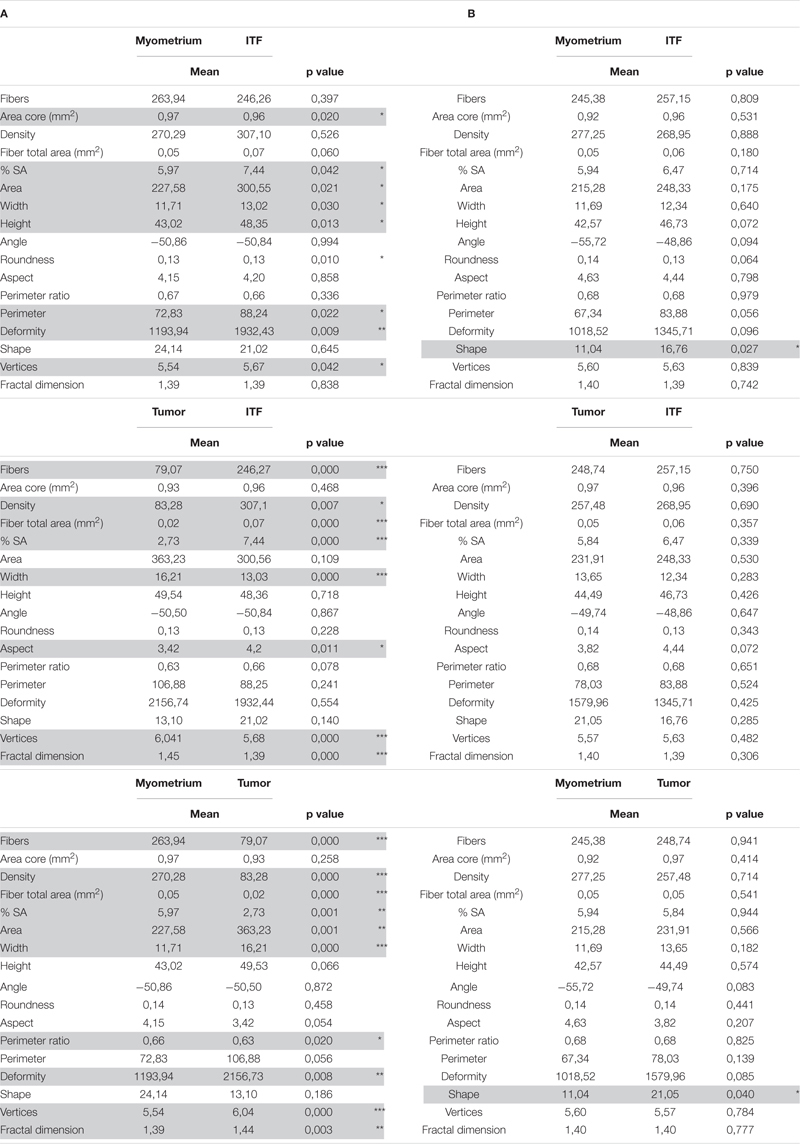

(A) Comparison of morphometric parameters obtained after reticular fiber segmentation between myometrium and ITF, tumor and ITF, and myometrium and tumor in 1 × 1-mm uADC ROIs. (B) Comparison of morphometric parameters obtained after reticular fiber segmentation between myometrium and ITF, tumor and ITF, and myometrium and tumor in 1 × 1-mm leiomyosarcoma (uLMS) ROIs. Fisher–Snedecor test and Student *T*-test were applied for statistical comparison. Mean, standard deviation (SD), p values, and significance (* < 0.05, ** < 0.01, and *** < 0.001) are shown. Total fibers, area of the core, and sum of the total area of the fibers were measured to calculate the density (number of fibers/mm^2^) and percentage of stained area (% SA). Area in μm^2^. Length and width in μm.

### Heterogeneous Immune Environment at the ITF

In order to characterize immune infiltrate at the ITF, we used six-color multiplex immunostaining (CD20, CD3, CD8, CD68, CK, and DAPI) to estimate four different immune infiltrate subpopulations in the same 5 × 4 mm ROIs of uADCs and uLMS, used for reticular fiber analysis in sequential cuts in large areas of uADCs and uLMS. The immune infiltrate was assessed in 95.8% of uADC and in 81% of uLMS (23 out of 24 and 9 out of 11, respectively) (Supplementary Table 1). Overall, approximately 1,595,232 cells were counted and evaluated by digital pathology. Although the uLMS had a lower number of total cells, the two tumor types displayed the same amount of total immune infiltrate (956 for uADC and 991 for uLMS) (Figure 3). However, we observed diverse immune cellular compositions at the ITF, finding a clearly heterogeneous distribution of B and T lymphocytes and macrophages in the different tumors analyzed. Comparing the two tumors, B lymphocytes (CD20^+^) appeared in lowest numbers out of the total immune population, being statistically smaller in uLMS, while there was a significant increase in macrophages, which represented the highest immune population in uLMS. Conversely, T lymphocytes (CD3^+^) emerged as the predominant immune infiltrate in uADC. The relative frequency of CD20, CD3, CD8, and CD68-positive cells within each tumor reflected that uADCs and uLMS exhibited cellular heterogeneity regarding immune distribution, whereas the total amount of infiltrate remained the same in 5 × 4-mm ITF (Figure 3).

Aiming for a more thorough characterization of the immune infiltrate, and given the heterogeneity observed, we proceeded to analyze the 1 × 1-mm ROIs, dividing the regions between tumor, myometrium, and ITF, choosing the same regions used for reticular fiber analysis (Supplementary Figure 1). After statistical comparison, we observed that uLMS had less total immune infiltrate than uADC (860 cells/mm^2^ vs. 276 cells/mm^2^) in the tumor areas, and in fact, fewer overall cells with markers were identified in uLMS (Figure 4); however, only CD8 became statistically smaller (132 cells/mm^2^ vs. 12 cells/mm^2^). This observation suggests that uLMS tumor areas are colder, or less abundant in terms of immune infiltrate, than uADC (Figure 4). Regarding uADC and uLMS myometrium, no significant differences in distribution of CD20-, CD3-, CD8-, and CD68-positive cells were found between the two tumor types, as expected, with a low number of total cells in the myometrium near uLMS (Figure 4). However, the 1 × 1-mm ITF regions displayed significant differences between CD20-, CD3-, and CD8-positive cells per mm^2^, being higher in uADC. The macrophage remained the most abundant existing cells in both ITF tumors (Figure 4). Interestingly, the 1-mm^2^ ITF ROIs results were more accurate than 5 × 4-mm ITF. The CD20 population still remained the lowest of all in both tumors, being significantly smaller in uLMS. While CD68-positive cells were the predominant population in uLMS, CD3 was the highest in uADC and again significantly different compared to uLMS.

### Transcriptional Profiling Reveals a Possible Role for Antimicrobial Peptides in Immune Response at the ITF of uADC

**FIGURE 5:**
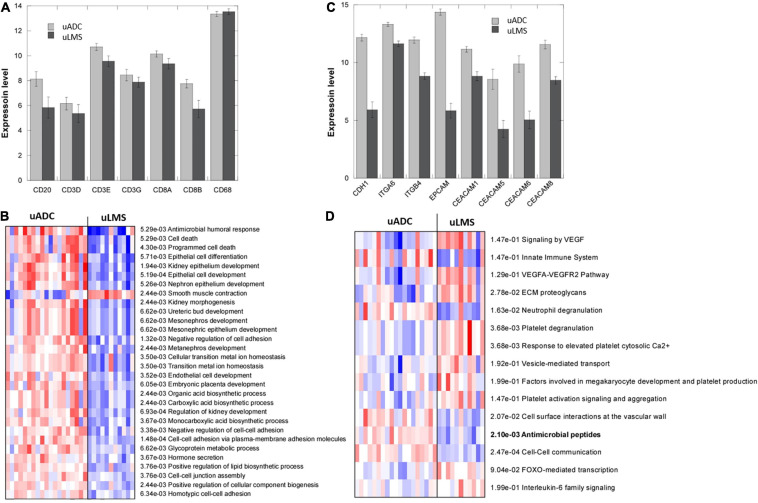
Transcriptomic profiling of invasive tumor front (ITF) in uterine adenocarcinomas (uADC) and leiomyosarcomas (uLMS). **(A)** RNA-seq expression levels (as counts per million, log2 scale) of cellular markers for macrophages (CD68), T cells (CD3 and CD8), and B lymphocytes (CD20). Slight upregulation of B and T cell markers is observed. **(B)** Parametric analysis of gene-set enrichment using the Gene Ontology biological process dataset. Red and blue indicate activated and suppressed pathways, respectively. **(C)** RNA-seq expression levels (as counts per million, log2 scale) of genes related to cell adhesion and epithelial cell development. Increased gene expression in uADC vs. uLMS was significant in all the markers. **(D)** Parametric analysis of gene-set enrichment using the curated reactome dataset reveals a possible role for antimicrobial peptides in immune response of uADC. Red and blue indicate activated and suppressed pathways, respectively.

Differential gene expression analyses revealed statistically significant upregulation of 142 genes and downregulation of 97 genes when comparing uADC (*n* = 19) vs. uLMS ITF (*n* = 11) (Supplementary Table 1). A slight upregulation of T cell and B cell markers was observed in uADC ITF, but no differences were detected in myeloid expression markers (Figure 5A) in accordance with the multiplex immunofluorescence findings. Parametric analysis of gene-set enrichment using GO showed the activation of several biological processes related to cell adhesion, epithelial cell development, and kidney morphogenesis in uADC ITF, likely resulting from their intrinsic tumor morphology (Figure 5B). Indeed, uADC ITF showed increased expression of E-cadherin, EPCAM, and several keratins and integrins (Figure 5C).

Interestingly, antimicrobial humoral response appeared to be activated in uADC ITF. By querying the curated reactome database, upregulation of antimicrobial peptides was also observed, along with innate immune response and neutrophil degranulation (Figure 5D). ECM proteoglycans seemed to be upregulated in the uLMS ITF, as expected given their mesenchymal phenotype.

### Genome-Wide DNA Methylation Analysis Identifies an Epigenetic Signature of ITF Related to Cell Adhesion and Extracellular Matrix

**FIGURE 6:**
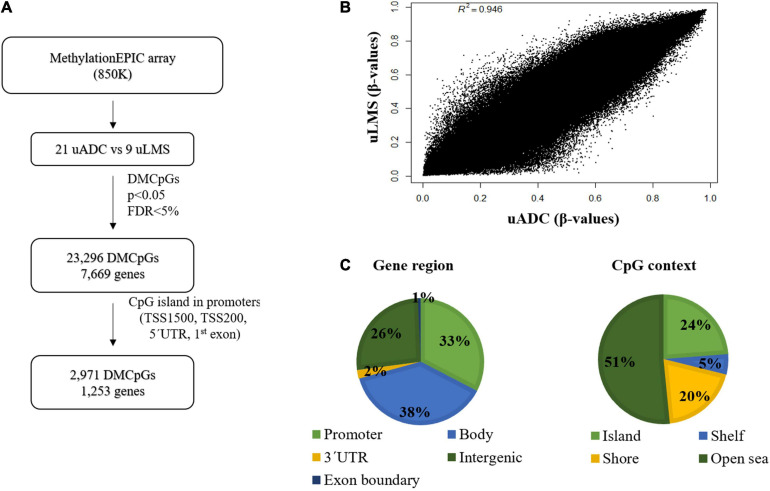
Genome-wide DNA methylation analysis in invasive tumor front (ITF) of primary uterine adenocarcinomas (uADC) and leiomyosarcomas (uLMS) shows a small proportion of CpGs with differential methylation pattern. **(A)** Schematic flowchart used to identify differentially methylated CpGs (DMCpGs) in primary ITF between uADC and uLMS. **(B)** Scatter plot representing mean normalized levels of DNA methylation (β-values) in primary ITF of uADC and uLMS. **(C)** Genomic distribution of 23,296 differentially methylated CpGs (DMCpGs) in primary ITF of uADC and uLMS, in relation to their respective location regarding CpG context and gene region.

**FIGURE 7:**
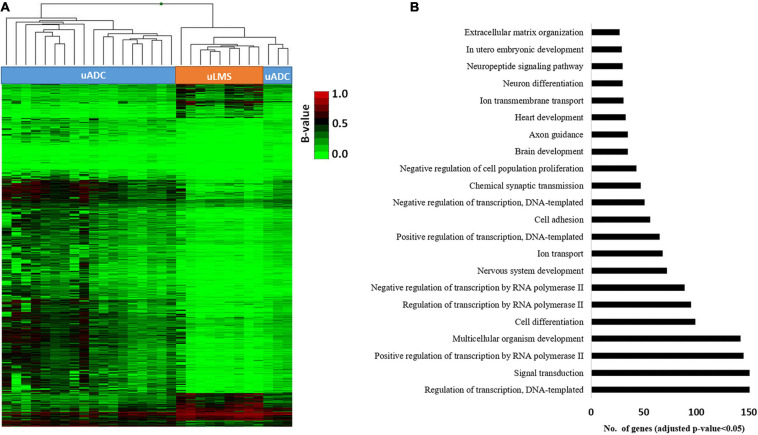
Identification of differential DNA methylation signature of promoter CGIs in invasive tumor front (ITF) of primary uterine adenocarcinomas (uADC) and leiomyosarcomas (uLMS). **(A)** Supervised hierarchical clustering of the most variable CpGs (2,971 CpGs and 1,253 genes; FDR < 5%) from island and promoter regions between the primary ITF of uADC and uLMS. **(B)** Gene ontology (GO) analysis of the biological process categories for the 1,253 differentially methylated genes at CpG island and promoters between the primary ITF of uADC and uLMS.

The DNA methylation profile of the primary ITF was compared in uADC (*n* = 21) and uLMS (*n* = 9) using the Infinium MethylationEPIC array (850 K) (Figure 6A). The scatter plot of this epigenomic comparison revealed that the ITFs of both tumor types share a similar general DNA methylation pattern (*R*^2^ = 0.946) (Figure 6B). In this sense, most of the 665,840 valid CpGs analyzed had similar DNA methylation levels, while a small proportion of CpGs (3.5%, 23,296 CpGs) showed significant differences (*p* < 0.05; FDR < 5%) between uADC and uLMS. These differentially methylated CpGs (DMCpGs) were distributed in several regions of the genome (Figure 6C), including CpG islands (CGIs) and promoters. We found an epigenetic signature of 2,971 DMCpGs (corresponding to 1,253 genes) located in CGIs of promoters, which were able to differentiate the ITF of uADC and uLMS (Figure 7A). Of note, the GO analysis of this epigenetic signature revealed enrichment of differentially methylated genes between uADC and uLMS related to cell adhesion and ECM organization pathways, among others. However, no evident enrichment of immune response pathways was detected (Figure 7B).

### Gene Expression Profiling and DNA Methylation Identify a 20-Gene Epigenetic Signature Characteristic of ITF

**FIGURE 8:**
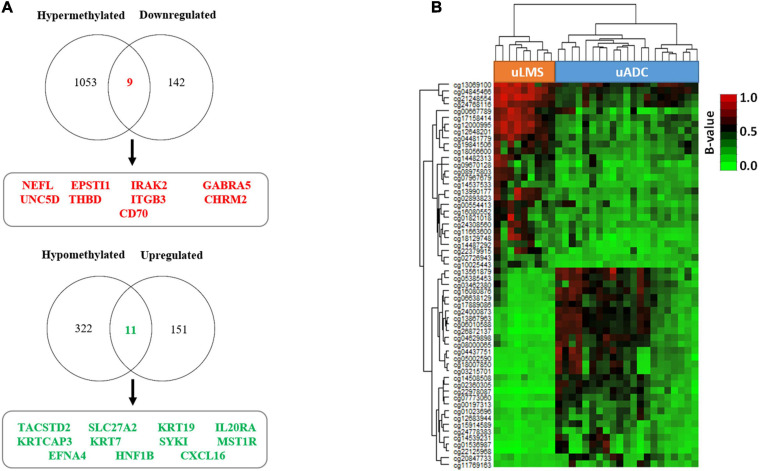
Invasive tumor front (ITF) is defined by a 20-gene expression signature, under epigenetic regulation, in primary uterine adenocarcinomas (uADC) and leiomyosarcomas (uLMS). **(A)** Venn diagrams showing the differentially methylated and differentially expressed genes between the primary ITF of uADC and uLMS. The names of the nine hypermethylated and downregulated genes in uADC compared to uLMS are indicated in red, and those of the 11 hypomethylated and upregulated genes in uADC compared to uLMS are represented in green. **(B)** Supervised hierarchical clustering of the CpGs from promoter CGIs (corresponding to 20 genes) whose methylation status correlated with significant changes in gene expression. β-Values represent the normalized levels of DNA methylation.

As hypermethylation of promoters has been demonstrated to induce gene silencing in several tumor types, including uterine neoplasms (Zhang et al., 2018), we decided to evaluate the association between the gene expression and methylation profiling of promoter CGIs previously found in the TIF of primary uterine uADC and uLMS. We found 20 genes whose methylation status correlated with significant changes in gene expression (Figure 8A and Supplementary Table 3); specifically, nine hypermethylated genes showed downregulated gene expression, while 11 hypomethylated genes were overexpressed. Of note, these 20 genes (corresponding to 58 CpGs) represent a signature able to clearly differentiate the ITF of primary uterine uADC and uLMS (Figure 8B). Importantly, some of the genes of this signature are related to immune system, cell adhesion, and tumor stroma.

## Discussion

In the uterus, uADC and uLMS account for a significant proportion of malignant tumors associated with distant metastasis. These tumors possess distinct morphological and molecular features, and also a differing cell of origin. However, both occur in the uterus, and each of them invades the myometrium as the initial step for tumor progression. Although dissimilar in many aspects, it is feasible that they share some adaptive responses while invading the myometrium, or alternatively, they could produce a similar response to tumor invasion in the myometrium.

The process of stromatogenesis refers to the formation of new stroma in the active sites of tumor invasion and metastasis. It is proposed that the formation of this new stroma is generated and governed by the interplay between tumor cells and the complicity of adjacent activated fibroblasts. This new stroma disrupts the normal structures of the ECM and its continuity and favors easy penetration by tumor cells, thus helping their migration and immune cell infiltration among other cells (Giatromanolaki et al., 2007; Horrée et al., 2007; Bremnes et al., 2011). The ECM composition and spatial organization of collagen fibers in the newly formed stroma is decisive not only for development of several neoplasia but for tumor prognosis (Kondo, 2011; Acerbi et al., 2015; Devendra et al., 2018; Xu et al., 2019). In fact, like type I collagen, reticulin distribution is increased in many cancers such as head and neck squamous cell cancer, breast, pancreas, and colorectal cancer (Nissen et al., 2019) and increases proliferation, migration, and metastasis in pancreatic and glioblastoma cancer cells as well as in invasive prostate cancer (Chintala et al., 1996; Menke et al., 2001; Kanematsu et al., 2004). Thus, in-depth characterization of morphometric features of reticular fibers (collagen type III) in invasive tumors could provide advanced insight into mechanisms underlying the invasion and galvanize targeted strategies for precision medicine (Kanner et al., 2013; Walke and Bhagat, 2017).

Although several aspects of the ITF have been characterized in other types of tumors, comparison between them is very complicated due to differing quantification methods. Furthermore, the exact size of the true invasion front is also under debate (Horrée et al., 2007) and little is known about the histomorphometry of the fibers at the ITF of uADC and uLMS. When analyzing the 5 × 4-mm ITF areas of uADC and uLMS, we found very few significant differences between morphometric parameters, indicating a striking similarity between the ITF of the two tumor types. In a restrictive analysis, choosing a uADC sample group with a more homogeneous architecture (based on subjective criteria), all the significant differences disappeared, suggesting that an inclusive analysis reflects the architectural heterogeneity of the uADC tumors. We also circumvented the issue of variable tissue amounts when analyzing 5 × 4-mm ITF ROIs by increasing the magnification of the ROIs to 1 mm^2^ within the previously studied 5 × 4-mm ROIs in which we ensured 50% each of the tumor and myometrium. After the necessary controls, we found that the area of the fibers in the 1-mm^2^ ITF was no longer significantly different between uADC and uLMS, but the differences in deformity remained, indicating the potential value of this parameter as a differential characteristic between the two types of tumors. We hypothesized that the area of the fibers in the 5 × 4-mm areas could vary due to an uneven contribution of the tumor to the analysis, given that the area was no longer different in the 1-mm^2^ ITF ROIs. The algorithm showed notably high sensitivity when measuring morphometric parameters, since in the 1-mm^2^ areas of myometrium we did not observe any significant differences between the two tumors, as expected. However, the large number of differences observed between 1-mm^2^ areas of pure tumor tissue reflects the ability to detect differences between negligible reticular fiber features of the tumors under subjective criteria.

At a microscopic level, ROIs of 1 mm^2^ were sufficient for comparison of reticular fiber morphometric parameters between the tumor, myometrium, and ITF of uADC and uLMS. Furthermore, 1-mm^2^ ROIs allowed us to characterize the microarchitecture of the reticular fibers within the same type of tumor. Interestingly, comparisons between tumor, myometrium, and ITF in uLMS revealed a high degree of similarity and little disruption of tissue architecture. Conversely, in uADC we observed a very high number of significantly different morphometric features comparing the abovementioned ROIs (Table 1), suggesting that continuity of ECM architecture is much more disrupted in uADC than uLMS tumors. Regardless of statistical difference, observing the mean of morphometric parameters at the ITF of uADC, we found that almost all values fell within the range of myometrium and tumor means, except for density (along with increased%SA) and aspect (linearization) of fibers (Table 1). Since the areas were carefully chosen to represent 50% each of myometrium and tumor components, we hypothesized that most changes observed at the uADC ITF could be attributable to the contribution of adjacent tissue. However, we cannot exclude the possibility of orchestrated ECM remodeling by tumor and non-tumoral cells to favor tumor cell migration (Giatromanolaki et al., 2007; Horrée et al., 2007; Bremnes et al., 2011). In fact, during tumor progression, cancer-associated fibroblasts are key players in dysregulated collagen turnover, leading to tumor fibrosis as evidenced by increased collagen depositions in the immediate periphery of the tumor (Pankova et al., 2016). Consistent with our results, we observed an increase of fiber density at the uADC ITF compared to tumor. In addition, the collagens are often cross-linked and linearized leading to stiffening of the tissue in several tumors (Nissen et al., 2019), which we also observed in uADC ITF as displayed by a significantly longer appearance (linearization) of the reticular fibers at the uADC ITF. ECM stiffening could in turn elicit behavioral effects on surrounding tumor cells regarding cell proliferation, differentiation, gene expression, migration, invasion, metastasis, and survival, all hallmarks of cancer (Pickup et al., 2014), and which correlate with worse patient outcomes patient (Leeming et al., 2011; Kehlet et al., 2016). Nevertheless, there are still several features of the fiber topology to take into account for future studies that would require smaller regions, such as analysis of fiber orientation with respect to the tumor–myometrium boundary, which would provide additional value for prognosis (Acerbi et al., 2015; Conklin et al., 2011; Xu et al., 2019).

Through multiplex cellular analyses, we could demonstrate that the uADC ITF contains a heterogeneous immune environment compared to the one in LSM. We found variance between different CD8 + T cell and macrophage subpopulations of different ITF in uADC and LSM. This suggests diverse myeloid and lymphocyte compositions of ITF immune environments, with CD8 + T cell and macrophage density possibly influenced by the presence of certain ECM components, such as collagens fibers, which could prevent interactions with other immune cells.

It has been shown that the reciprocal interaction between the ECM and tumor and non-tumor cells such as myofibroblasts determines recruitment, activation, and reprogramming of stromal, inflammatory, and immune cells (Sanegre et al., 2020). Despite the great similarity of reticulin scaffolding at the ITF of uADC and uLMS, we observed a differential immune infiltrate pattern, as informed by CD20, CD3, and CD8 markers. We thus cannot attribute a direct effect of reticulin architecture on the immune infiltrate composition right at the ITF. However, there could be a relationship between the morphometry of reticular fibers and the total amount of infiltrate, since the immune population is the same in both tumor types. A dense architectural barrier can be imposed not only by reticular fibers but also by ECM elements such as other collagens, hyaluronic acid, and laminins. Whereas high molecular weight hyaluronic acid provides structural integrity and suppresses the immune response by increasing regulatory T cell activity, laminins prevent transmigration and polarize leukocytes (Bollyky et al., 2009; Chanmee et al., 2016; Simon and Bromberg, 2017). ECM remodeling enzymes such as metalloproteinases, matrikines, and versicans act as cytokines and chemokines promoting IL expression and T cell chemoattractants, polarizing and activating the immune cells (Thomas et al., 2007; Hope et al., 2016). In fact, although an upregulation of collagen genes in uLMS tumors can be observed, it is still not clear whether these genes are responsible for immune density distribution. Another plausible explanation for leukocyte accumulation at the ITF of uADC could be that reticulin morphometric description suggests that uADC tumor areas seem stiffer than uLMS tumors, as indicated by the larger area and width and greater branching and fractal dimension of the fibers (although fewer fibers can be counted). Stiffer tumors could lead to a physical barrier, thus increasing the leukocyte concentration at the ITF. In addition, ECM stiffness can induce chromosomal rearrangements and transcriptional changes in several diseases (Simi et al., 2018; López-Carrasco et al., 2020) and many stiffness-sensitive genes may respond to stiffness in non-linear ways (Darnell et al., 2018).

Transcriptomic profiling of the ITF of uADC and uLMS suggests that activated neutrophils secreting antimicrobial peptides (AMP) may play a role in the immune response to oncogenesis in the context of endometrial tumors. Indeed, antimicrobial humoral response seems to be inactivated in uLMS, in stark contrast to uADC, in which there is an upregulation of this signature. AMPs have been described as important actors in angiogenesis and modulation of the immune response, via stimulation of chemokines and chemotaxis of leukocytes, and may also exert cytotoxic activity against tumor cells (Al-Rayahi and Sanyi, 2015; Jin and Weinberg, 2019). In fact, LCN2, one of the top upregulated genes in the ITF of uADCs (Supplementary Table 2), codes for neutrophil gelatinase-associated lipocalin, which has been reported to be associated with aggressive features of endometrial uADC (Mannelqvist et al., 2012; Cymbaluk-PA Oska et al., 2019) but is also described as an invasiveness and metastasis suppressor in other contexts (Lee et al., 2006; Lim et al., 2007; Tong et al., 2008; Santiago-Sánchez et al., 2020). Another highly upregulated antimicrobial peptide, DEFB1 (Supplementary Table 2), belongs to a family of cytotoxic peptides made by neutrophils. This gene has recently been identified as a chromosome 8p tumor-suppressor gene, downregulated in several malignancies such as salivary gland tumors, oral squamous cell carcinoma, and colon and liver cancer (Álvarez et al., 2018; Sun et al., 2019). Loss of orthologous murine defense-1 in mice enhances nickel sulfate-induced uLMS and causes mouse kidney cells to exhibit increased susceptibility to HPV-16 E6/7-induced neoplastic transformation (Sun et al., 2019). DEFB1 also induces formation of neutrophil extracellular traps (NETs) (Álvarez et al., 2018), mesh-like structures composed of cytosolic and granule proteins which are assembled on a scaffold of decondensed chromatin fibers in a process termed NETosis, which has been proposed to protect tumor cells from T cell- or natural killer (NK) cell-mediated toxicity (Teijeira et al., 2020). This should be further studied by developing a specific antibody panel to visualize and measure NET formation in tissue. Other upregulated antimicrobial genes such as the chemokine CXCL3 could contribute to recruitment of neutrophils at the ITF. Altogether, it is tempting to speculate that impaired antimicrobial humoral response could contribute to local or distant spread of uterine uLMS.

Epigenomic analysis showed a generally similar methylation pattern in the ITF of uADC and uLMS. However, when we focused our analysis on CGI promoters, we observed a different methylation profile in the ITF of the two tumor types. Interestingly, part of these differentially methylated genes was involved in biological processes related to cell adhesion and ECM organization. Of note, both biological pathways play a major role in invasion and migration of several types of tumors, including uterine neoplasms (Abal et al., 2007; Yadav et al., 2020), suggesting that epigenetic disruption of these pathways could influence the metastatic properties of uADC and uLMS. In addition, combining transcriptomic and epigenomic analysis, we observed an association between the methylation status of CGI promoters and expression in a set of 20 genes, implying epigenetic regulation of these genes in ITF. Importantly, this 20-gene epigenetic signature was able to clearly differentiate between ITF of uADC and uLMS. One of these genes was *ITGB3*, an integrin involved in cell adhesion and ECM whose deregulation is associated with cancer metastasis (Langsenlehner et al., 2006; Hu et al., 2018). Another epigenetically regulated gene identified in this signature was CXCL16, a chemokine-derived peptide implicated in antimicrobial and antitumoral response (Valdivia-Silva et al., 2015). Cancer patients with high levels of *CXCL16* expression have shown more elevated levels of CD4(+) and CD8(+) tumor-infiltrating lymphocytes and NK, leading to improved disease prognosis (Hojo et al., 2007; Valdivia-Silva et al., 2015). However, this chemokine can also induce invasion and metastasis via several mechanisms, such as the recruitment of mesenchymal stem cells into the tumor (Jung et al., 2013). In addition, CXCL16 contributes to ectopic endometrial stromal cell migration and invasion (Peng et al., 2019). We also detected other epigenetically regulated genes in this signature, including *CD70*, *IRAK*, *IL20RA*, *THBD*, *TACSTD2*, and *MST1R*, with implications related to the tumor immune microenvironment (Jain et al., 2014; Rossi et al., 2014; Babicky et al., 2019; Gu et al., 2020; Kumar et al., 2020; Gao et al., 2021). In our study, *TACSTD2* was overexpressed and hypomethylated in the ITF of uADC in contrast to uLMS. Interestingly, other studies have found highly expressed *TACSTD2* in aggressive endometrial carcinomas associated with tumor invasion (Bignotti et al., 2011), and it has been suggested as an attractive target for cancer immunotherapy in biologically aggressive endometrial carcinomas (Bignotti et al., 2012). Collectively, these data indicate that although the ITFs of uADC and uLMS have a similar global methylation status, there are also some significant epigenetic differences that could influence the invasive, migration, and tumor microenvironment properties of the two types of uterine neoplasms.

## Conclusion

In conclusion, our results demonstrate that uADC and uLMS share several morphometric and molecular features at the interface between tumor cells and myometrium, but also an intertumoral heterogeneity. There was a high degree of similarity between tumor and ITF in reticulin scaffolding, macrophage infiltration, and global DNA methylation pattern. However, uLMS had fewer T and B lymphocytes; additionally, neutrophil degranulation releasing antimicrobial peptides was observed in uADCs, but not in uLMS. Furthermore, gene expression profiling and DNA methylation identified a 20-gene epigenetic signature characteristic of ITF. It seems that the two tumors exhibit certain remarkable similarities when invading the myometrium despite being different neoplasms with profound morphologic and molecular differences, and originating from different precursor cells. These findings open up the possibility of investigating common therapeutic strategies based on these similarities in the ITF microenvironment. The results reported in this article have various potential clinical implications. The characteristics of the tumor microenvironment and immune surveillance in these two types of tumor suggest that targeting non-cancer cells, or mediators of their communication, may be efficient across different tumor types and could also complement other therapies. Moreover, tumor microenvironment features may provide insight into the mechanisms underlying the failure of previously effective anticancer drugs.

## Data Availability Statement

The datasets presented in this study can be found in online repositories. The names of the repository/repositories and accession number(s) can be found below: Methylation and expression profiling have been deposited at the NCBI GEO repository with accession numbers GSE171142 and GSE172034 respectively.

## Ethics Statement

The studies involving human participants were reviewed and approved by the Comité Coordinador de Ética de la Investigación Biomédica de Andalucía: Deciphering the site-specific tumor microenvironment of advanced uterine tumors (Biobank code: S1800086) and Comité de Ética de la Investigación with the codes CEIC-1892, CEIC-2083, and CEIC-1858. Written informed consent for participation was not required for this study in accordance with the national legislation and the institutional requirements.

## Author Contributions

RN and XM-G contributed to conception and design of the study. SS wrote the first draft of the manuscript. SS, NE, CA, JD-M, ÁD-L, EA, XM-G, and RN wrote sections of the manuscript. BD, RL, SR, EA, and XM-G provided the clinicopathological data. NE provided the collaborative consortium with microdissected samples. SS and RN designed and performed the morphometric characterization of the reticulin fibers. CA and DG performed the multiplex staining and analysis. JD-M, CS-A, and EA evaluated the transcriptomic profile. ÁD-L and MJ performed the genome-wide methylation analysis. All authors contributed to manuscript revision, read, and approved the submitted version.

## Conflict of Interest

The authors declare that the research was conducted in the absence of any commercial or financial relationships that could be construed as a potential conflict of interest.

## Footnotes

1 https://www.htgmolecular.com/assets/htg/publications/GL-HTG-EdgeSeq-Precision-Immuno-Oncology-Panel-GeneListAnnotated_01.pdf

2 http://ge-lab.org/idep/

## Supplementary Material

Supplementary Figure 1 In-depth characterization of the invasive tumor front (ITF) in uterine adenocarcinomas (uADC) and leiomyosarcomas (uLMS). A tumor biopsy from an adenocarcinoma (uADC) is shown. **(A)** Schematic pipeline for tumor ITF area selection for histomorphometric analysis, immune infiltrate, transcriptomics and methylation analysis. Scale bars represent 250 μm. **(B)** Selection from a 5 × 4 mm region of interest (ROI) of 1 × 1 mm ROIs of invasive tumor front (ITF), myometrium and tumor. The same criteria were applied for uADC and uLMS. For 5 × 4 mm image, scale bar represents 1 mm. For 1 × 1 mm images, scale bars represent 250 μm.

Supplementary Table 1 Case selection and regions of interest (ROIs) used in each characterization of uterine adenocarcinomas (uADC) and leiomyosarcomas (uLMS). Case identification is shown by tumor ID. The percentage of tumor/myometrium tissues for genomic and epigenetic profiling was evaluated in H&E stained samples. The total numbers of ROIs and tumors used for all the studies are shown. Invasive tumor front (ITF).

Supplementary Table 2 Transcript expression profile of the invasive tumor front (ITF) in uterine adenocarcinomas (uADC) and leiomyosarcomas (uLMS).

Supplementary Table 3 DNA methylation levels at promoter CGIs of 20 differentially methylated and differentially expressed genes associated with primary ITF in uterine adenocarcinomas (uADC) and leiomyosarcomas (uLMS).
