# Supplementary figures and images for: Characterizing the Invasive Tumor Front of Aggressive Uterine Adenocarcinoma and Leiomyosarcoma
Source: Front Cell Dev Biol. 2021 Jun 3;9:670185. doi: 10.3389/fcell.2021.670185 (PMC8209546; doi:10.3389/fcell.2021.670185)

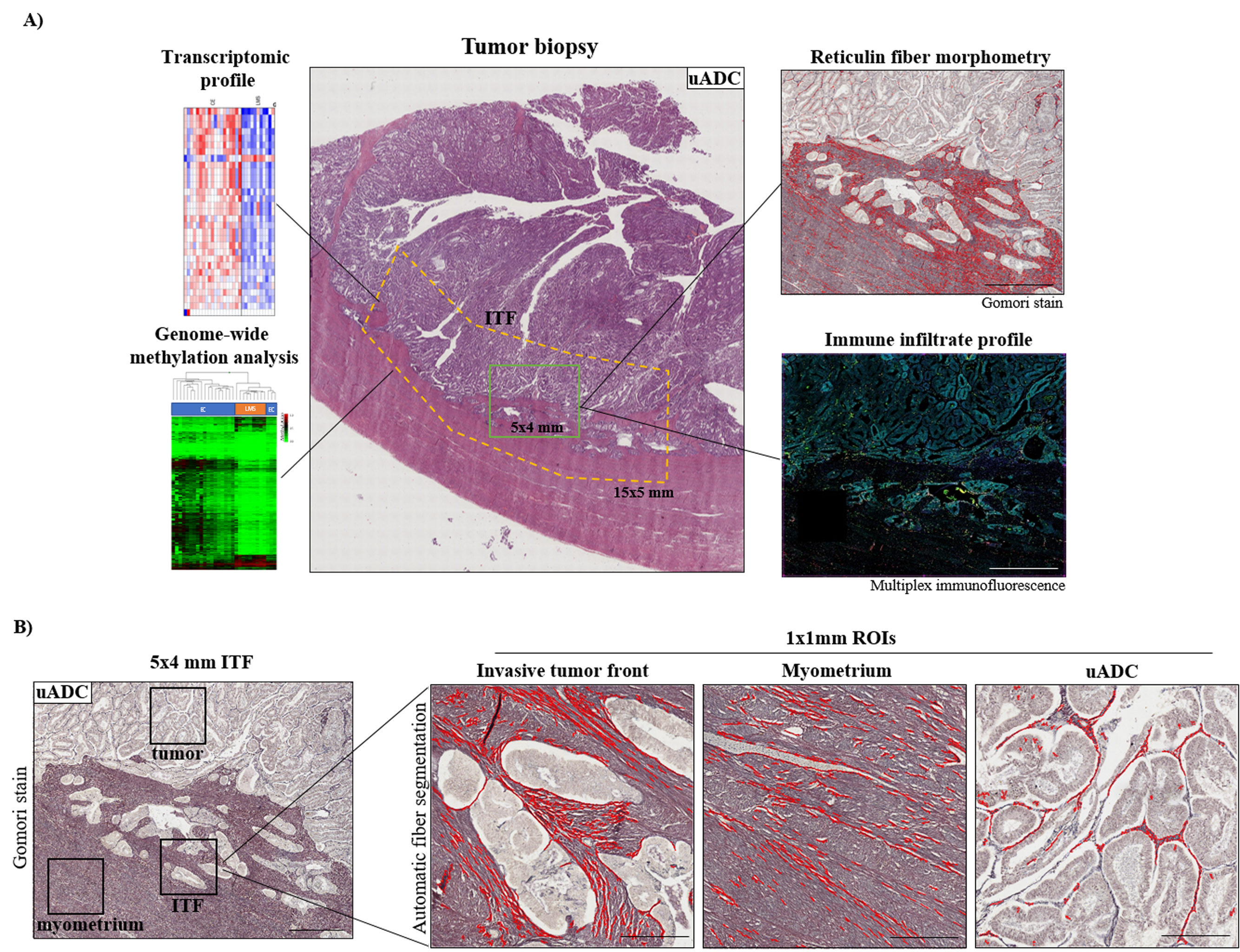

Supplement: Supplementary Figure 1 — In-depth characterization of the invasive tumor front (ITF) in uterine adenocarcinomas (uADC) and leiomyosarcomas (uLMS). A tumor biopsy from an adenocarcinoma (uADC) is shown. (A) Schematic pipeline for tumor ITF area selection for histomorphometric analysis, immune infiltrate, transcriptomics and methylation analysis. Scale bars represent 250 μm. (B) Selection from a 5 × 4 mm region of interest (ROI) of 1 × 1 mm ROIs of invasive tumor front (ITF), myometrium and tumor. The same criteria were applied for uADC and uLMS. For 5 × 4 mm image, scale bar represents 1 mm. For 1 × 1 mm images, scale bars represent 250 μm. [file Image_1.jpg]
