# Supplementary material for: Characterizing the Invasive Tumor Front of Aggressive Uterine Adenocarcinoma and Leiomyosarcoma
Source: Front Cell Dev Biol. 2021 Jun 3;9:670185. doi: 10.3389/fcell.2021.670185 (PMC8209546; doi:10.3389/fcell.2021.670185)
Supplement: Supplementary Table 3 — DNA methylation levels at promoter CGIs of 20 differentially methylated and differentially expressed genes associated with primary ITF in uterine adenocarcinomas (uADC) and leiomyosarcomas (uLMS). [file Table_3.docx]

| **Target ID** | **Chr** | **Position^1^** | **Gene name** | **uADC (mean)** | **uLMS (mean)** | **ADC-LMS**  **(mean differences)** | **FDR^2^** |
| --- | --- | --- | --- | --- | --- | --- | --- |
| cg18056600 | 17 | 4642047 | CXCL16 | 0.23 | 0.48 | -0.25 | 0.021 |
| cg19841506 | 17 | 4642056 | CXCL16 | 0.27 | 0.50 | -0.24 | 0.031 |
| cg24778383 | 19 | 6590895 | CD70 | 0.19 | 0.07 | 0.12 | 0.027 |
| cg15914589 | 3 | 10206523 | IRAK2 | 0.24 | 0.07 | 0.16 | 0.004 |
| cg18007850 | 3 | 10206537 | IRAK2 | 0.34 | 0.06 | 0.28 | 0.007 |
| cg03215701 | 3 | 10206542 | IRAK2 | 0.33 | 0.05 | 0.28 | 0.003 |
| cg04437751 | 3 | 10206628 | IRAK2 | 0.38 | 0.08 | 0.30 | 0.001 |
| cg05002590 | 3 | 10206633 | IRAK2 | 0.35 | 0.07 | 0.28 | 0.021 |
| cg11769163 | 20 | 23030102 | THBD | 0.21 | 0.09 | 0.12 | 0.027 |
| cg01023696 | 8 | 24813003 | NEFL | 0.27 | 0.12 | 0.15 | 0.019 |
| cg07773060 | 8 | 24813046 | NEFL | 0.30 | 0.08 | 0.22 | 0.003 |
| cg00197313 | 8 | 24813120 | NEFL | 0.26 | 0.05 | 0.21 | 0.001 |
| cg02360305 | 8 | 24813182 | NEFL | 0.35 | 0.06 | 0.29 | 0.004 |
| cg24000873 | 8 | 24813653 | NEFL | 0.42 | 0.08 | 0.34 | 0.001 |
| cg16080876 | 8 | 24813866 | NEFL | 0.44 | 0.14 | 0.30 | 0.005 |
| cg22978087 | 8 | 24814126 | NEFL | 0.33 | 0.02 | 0.31 | 0.002 |
| cg03462380 | 15 | 27112902 | GABRA5 | 0.40 | 0.17 | 0.23 | 0.048 |
| cg14508508 | 15 | 27113322 | GABRA5 | 0.33 | 0.11 | 0.22 | 0.008 |
| cg04845466 | 2 | 27665079 | KRTCAP3 | 0.43 | 0.78 | -0.36 | 0.001 |
| cg24768116 | 2 | 27665128 | KRTCAP3 | 0.38 | 0.70 | -0.33 | 0.001 |
| cg12000995 | 2 | 27665139 | KRTCAP3 | 0.24 | 0.73 | -0.48 | 0.001 |
| cg12648201 | 2 | 27665141 | KRTCAP3 | 0.22 | 0.71 | -0.49 | 0.001 |
| cg21248554 | 2 | 27665150 | KRTCAP3 | 0.39 | 0.81 | -0.42 | 0.001 |
| cg17158414 | 2 | 27665306 | KRTCAP3 | 0.26 | 0.69 | -0.43 | 0.001 |
| cg05385453 | 8 | 35092683 | UNC5D | 0.44 | 0.13 | 0.31 | 0.008 |
| cg13561879 | 8 | 35092687 | UNC5D | 0.47 | 0.20 | 0.28 | 0.043 |
| cg08000065 | 8 | 35092823 | UNC5D | 0.38 | 0.12 | 0.25 | 0.011 |
| cg26872137 | 8 | 35092870 | UNC5D | 0.40 | 0.04 | 0.35 | 0.001 |
| cg06010588 | 8 | 35092876 | UNC5D | 0.40 | 0.08 | 0.33 | 0.002 |
| cg13867963 | 8 | 35092878 | UNC5D | 0.42 | 0.07 | 0.36 | 0.002 |
| cg17889086 | 8 | 35093161 | UNC5D | 0.36 | 0.12 | 0.24 | 0.006 |
| cg06638129 | 8 | 35093176 | UNC5D | 0.42 | 0.15 | 0.27 | 0.012 |
| cg04629898 | 8 | 35093210 | UNC5D | 0.41 | 0.09 | 0.31 | 0.003 |
| cg14487292 | 17 | 36105517 | HNF1B | 0.13 | 0.34 | -0.21 | 0.034 |
| cg22379915 | 17 | 39684287 | KRT19 | 0.12 | 0.40 | -0.28 | 0.031 |
| cg02893823 | 17 | 39684544 | KRT19 | 0.17 | 0.49 | -0.32 | 0.024 |
| cg13990177 | 17 | 39684563 | KRT19 | 0.21 | 0.52 | -0.31 | 0.008 |
| cg14539231 | 13 | 43566262 | EPSTI1 | 0.25 | 0.07 | 0.18 | 0.012 |
| cg22125968 | 13 | 43566472 | EPSTI1 | 0.20 | 0.03 | 0.17 | 0.008 |
| cg01536987 | 13 | 43566492 | EPSTI1 | 0.22 | 0.04 | 0.18 | 0.016 |
| cg12683944 | 17 | 45330929 | ITGB3 | 0.24 | 0.10 | 0.14 | 0.039 |
| cg18129748 | 3 | 49941408 | MST1R | 0.11 | 0.45 | -0.34 | 0.011 |
| cg11663600 | 3 | 49941416 | MST1R | 0.14 | 0.41 | -0.26 | 0.019 |
| cg24308560 | 3 | 49941425 | MST1R | 0.18 | 0.41 | -0.23 | 0.039 |
| cg02726943 | 15 | 50475039 | SLC27A2 | 0.12 | 0.24 | -0.12 | 0.019 |
| cg07967679 | 12 | 52626814 | KRT7 | 0.13 | 0.44 | -0.31 | 0.024 |
| cg08975803 | 12 | 52626818 | KRT7 | 0.17 | 0.42 | -0.25 | 0.009 |
| cg14482313 | 12 | 52626889 | KRT7 | 0.21 | 0.51 | -0.30 | 0.009 |
| cg14537533 | 12 | 52626904 | KRT7 | 0.15 | 0.32 | -0.17 | 0.014 |
| cg09670128 | 12 | 52627047 | KRT7 | 0.14 | 0.40 | -0.26 | 0.048 |
| cg00667789 | 1 | 59042065 | TACSTD2 | 0.23 | 0.66 | -0.43 | 0.007 |
| cg00554413 | 1 | 59042113 | TACSTD2 | 0.21 | 0.46 | -0.26 | 0.021 |
| cg16080552 | 1 | 59043199 | TACSTD2 | 0.20 | 0.41 | -0.22 | 0.009 |
| cg01821018 | 1 | 59043280 | TACSTD2 | 0.17 | 0.42 | -0.25 | 0.048 |
| cg10025443 | 9 | 93564339 | SYK | 0.09 | 0.31 | -0.23 | 0.001 |
| cg20847733 | 7 | 136554160 | CHRM2 | 0.28 | 0.10 | 0.18 | 0.039 |
| cg04481779 | 6 | 137366125 | IL20RA | 0.25 | 0.64 | -0.38 | 0.001 |
| cg13069100 | 1 | 155035008 | EFNA4 | 0.51 | 0.75 | -0.25 | 0.043 |

^1^Position according to GRCh37/hg19 (UCSC Genome Browser); ^2^False discovery rate (FDR).
